# A Novel Framework for Synthesizing Biosensor Data and the Minimum Data Set to Improve Alzheimer's Care

**DOI:** 10.1002/alz70858_106806

**Published:** 2025-12-26

**Authors:** Brian B Johnson

**Affiliations:** ^1^ University of Minnesota, Minneapolis, MN, USA

## Abstract

**Background:**

The Minimum Data Set (MDS) is a tool developed by the Centers for Medicare & Medicaid Services (CMS) to standardize assessment for facilitating care management in long‐term care facilities. The integration of biosensor data into the assessment could provide clinicians with real‐time and longitudinal information that allow for an unprecedented insight into the physical and cognitive health of the individual.

**Method:**

An example of an area where biosensor data could be integrated into the MDS in Section G0300. Balance During Transitions and Walking. Currently, the assessment is based on eye‐witness of the clinician and uses the following coding scores. 0. Steady at all times.1. Not steady, but able to stabilize without staff assistance. 2. Not steady, only able to stabilize with staff assistance. 8. Activity did not occur. For the following activities A. Moving from seated to standing position. B. Walking (with assistive device if used). C. Turning around and facing the opposite direction while walking. D. Moving on and off toilet. E. Surface‐to‐surface.

An individual using a biosensor for gait analysis could both reduce the amount of time for assessment and provide more accurate and objective information. Continuous monitoring is feasible with this approach along with more sophisticated detection of movement and gait anomalies. Several solutions may be appropriate for further research including inertial measurement units, pressure‐sensitive insoles, radar‐based monitoring, and depth‐sensing cameras.

**Result:**

The biosensing method combined with MDS codes could utilize the following data driven criteria. 0. No significant deviation in step symmetry, sway velocity, or transition stability. 1. Minor deviations detected but corrected within a threshold time (e.g., <1.5s delay in stability) 2. High sway, excessive corrective steps, prolonged stabilization time (>2s). 8. No sensor data recorded for the movement.

**Conclusion:**

Integrating gait analysis sensors into MDS Section G0300 would enhance assessment accuracy, reduce subjectivity, and provide real‐time fall risk insights. This could be particularly beneficial in skilled nursing facilities, rehabilitation centers, and fall prevention programs.

